# LncRNAs are altered in lung squamous cell carcinoma and lung adenocarcinoma

**DOI:** 10.18632/oncotarget.13651

**Published:** 2016-11-26

**Authors:** Bing Liu, Yifei Chen, Jiong Yang

**Affiliations:** ^1^ Department of Respiratory Medicine, Zhongnan Hospital of Wuhan University, Wuhan, China

**Keywords:** lung squamous cell carcinoma, lung adenocarcinoma, long non-coding RNA, overall survival, gene regulation

## Abstract

Long non-coding RNAs (lncRNAs) have been implicated in pathogenesis of various cancers, including lung squamous cell carcinoma (LUSC) and lung adenocarcinoma (LUAD). We used cBioPortal to analyze lncRNA alteration frequencies and their ability to predict overall survival (OS) using 504 LUSC and 522 LUAD samples from The Cancer Genome Atlas (TCGA) database. In LUSC, 624 lncRNAs had alteration rates > 1% and 64 > 10%. In LUAD 625 lncRNAs had alteration rates > 1% and 36 > 10%. Among those, 620 lncRNAs had alteration frequencies > 1% in both LUSC and LUAD, while 22 were LUSC-specific and 23 were LUAD-specific. Twenty lncRNAs had alteration frequencies > 10% in both LUSC and LUAD, while 44 were LUSC-specific and 16 were LUAD specific. Genome ontology and pathway analyses produced similar results for LUSC and LUAD. Two lncRNAs (IGF2BP2-AS1 and DGCR5) correlated with better OS in LUSC, and three (MIR31HG, CDKN2A-AS1 and LINC01600) predicted poor OS in LUAD. Chip-seq and luciferase reporter assays identified potential IGF2BP2-AS1, DGCR5 and LINC01600 promoters and enhancers. This study presented lncRNA landscapes and revealed differentially expressed, highly altered lncRNAs in LUSC and LUAD. LncRNAs that act as oncogenes and lncRNA-regulating transcription factors provide novel targets for anti-lung cancer therapeutics.

## INTRODUCTION

Lung cancers cause the most cancer deaths worldwide, and 5-year overall survival rates remain below 15% [1, 2]. Lung cancers include non-small cell (NSCLC) and small cell lung cancer (SCLC), with more than 80% of lung cancer patients suffering from NSCLC [1]. Lung squamous carcinoma (LUSC) and lung adenocarcinoma (LUAD) are the most common NSCLCs [3]. Non-coding RNAs, transcriptionally active genes that do not code for proteins, account for more than 80% of all genes. Long non-coding RNAs (lncRNAs) are non-coding RNAs longer than 200 nucleotides [4]. They regulate many normal and disease-related cellular functions, including cancer-related processes, and may predict overall survival in some type of cancers [5].

Many lncRNAs are biomarkers in lung cancer. Metastasis-associated lung adenocarcinoma transcript 1 (MALAT-1) may promote lung cancer metastasis to the brain via epithelial-mesenchymal transition, and was both highly expressed and associated with poor prognosis in NSCLC [6]. PVT1 was upregulated in NSCLC tissues, was associated with tumor stage and local metastasis, and is a potential therapeutic target [7]. HOTAIR was highly expressed in advanced stage lung cancers. It might promote cancer cell migration and aggression, and was correlated with metastasis and poor prognosis [8, 9]. ANRIL was also upregulated in NSCLC tumor tissues, and was positively associated with tumor stage, size and metastasis [10, 11]. Although many studies have associated lncRNAs with NSCLC, most have involved small sample sizes, with little insight into lncRNA mechanisms of action and prognostic values. The Cancer Genome Atlas (TCGA) is a public database (http://cancergenome.nih.gov/) that includes 33 cancer types, along with related clinical and genomic data. The cBioPortal (http://cBioPortal.org) is a web-based public tool for exploring, visualizing, and analyzing TCGA and other cancer genetic data [12, 13]. In this study, we used the TCGA database and cBioPortal tool to analyze lncRNA alterations and survival prediction values in both LUSC and LUAD. We also assessed the *Gene* ontologies (*GO*) and important pathways associated with these selected lncRNAs and their coexpressed genes. Potential promoters and enhancers of the lncRNAs, IGF2BP2-AS1, DGCR5, and LINC01600 were identified and analyzed (Supplementary Figure S1).

## RESULTS

### LncRNAs were highly altered in LUSC and LUAD

**Figure 1:**
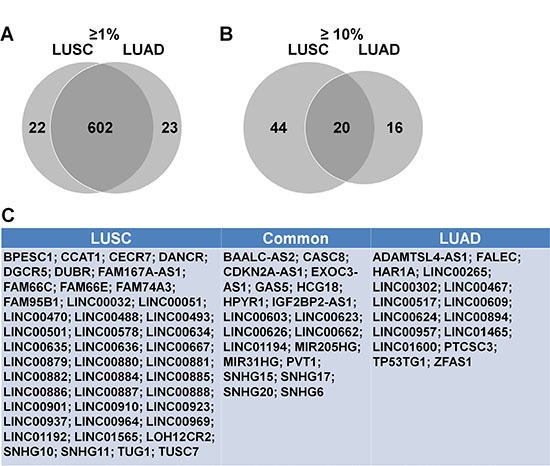
Highly altered lncRNAs in LUSC and LUAD Venn analysis of lncRNAs with alteration frequencies > 1% (**A**) This included 647 total lncRNAs, with 602 shared between LUSC and LUAD. Venn analysis of lncRNAs with alteration frequencies > 10% (**B**) This included 64 and 36 lncRNAs in LUSC and LUAD, respectively, with 20 shared. LncRNAs with alteration frequencies > 10% (**C**) Left box, altered only in LUSC; middle box, altered in both LUSC and LUAD; right box, altered only in LUAD.

To identify lncRNAs with high alteration frequencies in LUSC and LUAD, we downloaded the current lncRNA database from HUGO (www.HUGO.org), which includes 2772 lncRNAs (Supplementary Table S1). 2745 of these were recognized in cBioPortal (Supplementary Table S2), and their alternation frequencies were assessed in the LUSC and LUAD datasets. 624 and 64 lncRNAs had alteration rates > 1% and > 10%, respectively, in LUSC (Supplementary Table S3, Table 1). 625 and 36 lncRNAs had alteration rates > 1% and > 10%, respectively, in LUAD (Supplementary Table S4, Table 2). A *venn* diagram was used to determine commonly and differentially altered lncRNAs. In a venn diagram analysis, 620 of those lncRNAs with alteration frequencies > 1% were commonly altered in both LUSC and LUAD (Figure 1A), 22 lncRNAs were LUSC-specific and 23 were LUAD-specific. Of the lncRNAs with alteration frequencies > 10%, 20 were commonly altered, 44 were LUSC-specific and 16 were LUAD-specific (Figure 1B–1C).

**Table 1:** LncRNAs with alteration frequency higher than 10% in LUSC

LncRNA	Alteration	Alteration reason	Cytoband	Position
IGF2BP2-AS1	0.54	Amp; high exp	p26.3	Ch3:185,712,528–185,729,787
LINC00888	0.46	Amp	p26.3	Ch3:183,447,608–183,456,013
LINC00501	0.44	Amp	p26.3	Ch3:177,294,442–177,323,418
LINC00578	0.44	Amp	p26.3	Ch3:177,441,921–177,752,305
LINC00887	0.40	Amp; high exp	p26.3	Ch3:194,296,465–194,312,803
LINC00884	0.38	Amp	p26.3	Ch3:194,487,454–194,518,279
LINC00969	0.38	Amp	p26.3	Ch3:195,658,062–195,739,964
LINC00885	0.38	Amp	p26.3	Ch3:196,142,636–196,160,890
LINC01192	0.35	Amp	p26.3	Ch3:163,127,923–163,361,563
EXOC3-AS1	0.33	Amp; high exp	p15.33	Ch5:441,498–443,160
LINC00880	0.29	Amp	p26.3	Ch3:157,081,667–157,123,004
LINC00881	0.29	Amp	p26.3	Ch3:157,089,881–157,101,135
CDKN2A-AS1	0.29	Del	p24.3	Ch9:21,966,929–21,967,751
LINC00886	0.28	Amp	p26.3	Ch3:156,747,346–156,817,062
PVT1	0.23	High exp; amp	p23.3	Ch8:127,794,533–128,101,253
BPESC1	0.2	Amp; high exp	p26.3	Ch3:139,104,185–139,125,171
DUBR	0.18	Amp; high exp	p26.3	Ch3:107,240,692–107,326,964
LINC00635	0.18	Amp; high exp	p26.3	Ch3:107,840,228–107,882,000
LINC01194	0.18	Amp	p15.33	Ch5:12,574,857–12,804,363
MIR31HG	0.17	Del; high exp	p24.3	Ch9:21,455,642–21,559,669
TUG1	0.16	Amp; del	p13	Ch22:30,970,677–30,979,395
LINC01565	0.16	Amp; high exp	p26.3	Ch3:128,572,000–128,576,086
CASC8	0.16	Amp; high exp	p23.3	Ch8:127,289,817–127,482,139
LINC00603	0.15	Amp	p15.33	Ch5:40,052,291–40,053,324
TUSC7	0.14	Amp; high exp	p26.3	Ch3:116,709,235–116,723,581
LINC00964	0.14	High exp; amp	p23.3	Ch8:124,848,737–124,954,328
LINC00623	0.13	High exp; amp	p36.33	Ch1:120,913,275–121,009,291
SNHG20	0.13	Amp; high exp	p13.3	Ch17:77,086,716–77,094,990
SNHG11	0.13	High exp	p13	Ch20:38,446,578–38,450,921
LINC00882	0.13	Amp; high exp	p26.3	Ch3:106,836,811–107,240,641
LINC00879	0.13	Amp	p26.3	Ch3:94,938,172–95,152,509
HPYR1	0.13	Amp; high exp	p23.3	Ch8:132,560,498–132,561,479
LINC00626	0.12	High exp; amp	p36.33	Ch1:168,786,939–168,792,886
MIR205HG	0.12	High exp; amp	p36.33	Ch1:209,428,820–209,432,838
FAM66C	0.12	High exp; amp	p13.33	Ch12:8,180,209–8,216,151
DGCR5	0.12	High exp; amp	p13	Ch22:18,970,514–19,031,242
LINC00488	0.12	Amp; high exp	p26.3	Ch3:109,178,165–109,185,257
FAM167A-AS1	0.12	Del; high exp	p23.3	Ch8:11,368,402–11,438,658
FAM95B1	0.12	high exp; amp	p24.3	Ch9:40,321,299–40,329,221
GAS5	0.11	high exp	p36.33	Ch1:173,863,900–173,868,882
LINC00937	0.11	High exp; amp	p13.33	Ch12:8,295,986–8,396,803
SNHG10	0.11	High exp;	p13	Ch14:95,532,297–95,534,872
LINC00662	0.11	High exp; amp	p13.3	Ch19:27,684,580–27,793,940
SNHG17	0.11	High exp;	p13	Ch20:38,420,588–38,435,353
CECR7	0.11	High exp;	p13	Ch22:17,036,570–17,060,825
SNHG15	0.11	High exp;	p22.3	Ch7:44,983,023–44,986,961
FAM74A3	0.11	High exp;	p24.3	Ch9:66,976,520–66,976,991
LOH12CR2	0.1	High exp; amp	p13.33	Ch12:12,355,406–12,357,067
LINC00923	0.1	High exp; amp	p13	Ch15:97,572,185–97,874,550
LINC00470	0.1	High exp; amp	p11.32	Ch18:1,254,383–1,408,344
LINC00667	0.1	High exp;	p11.32	Ch18:5,237,826–5,246,508
LINC00493	0.1	High exp;	p13	Ch20:18,567,347–18,569,563
LINC00634	0.1	Amp	p13	Ch22:41,952,165–41,958,933
LINC00636	0.1	Amp	p26.3	Ch3:107,883,248–107,928,907
LINC00901	0.1	Amp	p26.3	Ch3:116,921,431–116,932,238
DANCR	0.1	High exp; amp	p16.3	Ch4:52,712,404–52,720,351
HCG18	0.1	High	p25.3	Ch6:30,287,397–30,327,150
BAALC-AS2	0.1	High amp	p23.3	Ch8:103,132,963–103,141,475
CCAT1	0.1	Amp	p23.3	Ch8:127,207,866–127,219,088
LINC00051	0.1	Amp; high exp	p23.3	Ch8:142,198,356–142,209,003
SNHG6	0.1	High amp	p23.3	Ch8:66,921,684–66,926,398
FAM66E	0.1	Del; high exp	p23.3	Ch8:7,955,014–8,008,755
LINC00032	0.1	Del; amp; high exp	p24.3	Ch9:27,245,684–27,282,793
LINC00910	0.1	High	p13.3	h17:43,369,845–43,389,199

**Table 2:** LncRNAs with alteration frequency higher than 10% in LUAD

LncRNA	Alteration	Alteration reason	Cytoband	Position
EXOC3-AS1	0.29	Amp; high exp	p15.33	Chr5:441498–443160
PVT1	0.25	Amp; high exp	q24.21	Chr8:127794533–128101253
LINC00623	0.21	Amp; high exp	p11.2	Chr1:120912238–121009291
CDKN2A-AS1	0.2	Del	p21.3	chr9:21966929–21967754
LINC00467	0.18	Amp; high exp	q32.2	Chr1:211382755–211435333
HCG18	0.15	High exp	p22.1	Chr6:30287397–30327156
FALEC	0.14	Amp	q21.3	Chr1:150515757–150518032
LINC01194	0.14	Amp	p15.2	Chr5:12574857–12805183
MIR31HG	0.14	Del	p21.3	Chr9:21454268–21559698
LINC00624	0.13	Amp	q21.2	Chr1:147258885–147517875
ADAMTSL4-AS1	0.13	Amp	q21.3	Chr1:150560202–150574552
LINC00302	0.13	Amp	q21.3	Chr1:152655429–152656805
LINC00609	0.13	Amp	q13.2	Chr14:36070427–36165288
PTCSC3	0.13	Amp	q13.2	Chr14:36135710–36176651
LINC00662	0.13	High exp	q11	Chr19:27684580–27793940
LINC00957	0.13	Amp; high exp	p13	Chr7:44039049–44044296
LINC00626	0.12	Amp; high exp	q24.2	Chr1:168786939–168792886
GAS5	0.12	Amp; high exp	q25.1	Chr1:173863900–173868882
SNHG15	0.12	Amp; high exp	p13	Chr7:44983023–44986961
BAALC-AS2	0.12	Amp; high exp	q22.3	Chr8:103132963–103141475
LINC00517	0.11	Amp	q21.1	Chr14:37896060–37902372
SNHG20	0.11	High exp	q25.3	Chr17:77086716–77094990
HAR1A	0.11	Amp; high exp	p13	Chr20:63102205–63104386
LINC00603	0.11	Amp	p13.1	Chr5:40052291–40053324
LINC00265	0.11	Amp; high exp	p14.1	Chr7:39733632–39793092
CASC8	0.11	Amp	q24.13	Chr8:127289817–127482139
HPYR1	0.11	Amp; high exp	q24.22	Chr8:132560498–132561479
LINC01465	0.1	High exp	q14.1	Chr12:62601751–62603690
SNHG17	0.1	High exp	q12	Chr20:38420588–38435353
ZFAS1	0.1	High exp	q13.13	Chr20:49278178–49295738
TP53TG1	0.1	High exp	q21.12	Chr7:87325225–87345515
SNHG6	0.1	High exp	q13.1	Chr8:66921684–66926398
LINC00894	0.1	Amp; high exp	q28	ChrX:149938628–150224580
IGF2BP2-AS1	0.1	High exp	p26.3	Chr3:185712528–185729787
LINC01600	0.1	High exp	p25.3	Chr6:2,621,913–2634603
MIR205HG	0.1	Amp; high exp	p36.33	Chr1:209428820–209432838

Genetic analysis showed that lncRNAs with alteration frequencies > 10% in LUSC accumulated within several cytobands. 22 lncRNAs were in one cytoband (chr 3: p26.3), and 10 were in a second cytoband (Ch8: p23.3) (Table 3). There was no such cytoband accumulation phenomenon for LUAD lncRNAs. The 9 lncRNAs in chromosome 1, and 5 lncRNAs in chromosome 8 were distributed in different cytobands (Table 4).

**Table 3:** Character of lncRNAs in LUSC

Chromosome	Cytoband	LncRNA
Chr 1	Chr1:p36.33	LINC00623; LINC00626; GAS5; MIR205HG
Chr 3	Chr3:p26.3	LINC00882; DUBR; LINC00635; LINC00636; LINC00488; TUSC7; LINC00901; LINC01565; BPESC1; LINC00886; LINC00880; LINC00881; LINC01192; LINC00501; LINC00578; LINC00888; IGF2BP2-AS1; LINC00887; LINC00884; LINC00969; LINC00885; LINC00879
Chr 4	Chr4:p16.3	DANCR
Chr 5	Chr5:p15.33	LINC01194; LINC00603; EXOC3-AS1
Chr 6	Chr6:p25.3	HCG18
Chr 7	Chr7:p22.3	SNHG15
Chr 8	Chr8:p23.3	BAALC-AS2; FAM167A-AS1; LINC00964; CCAT1; CASC8; PVT1; HPYR1; LINC00051; SNHG6; FAM66E
Chr 9	Chr9:p24.3	MIR31HG; CDKN2A-AS1; LINC00032; FAM95B1; FAM74A3
Chr 12	Chr12:p13.33	LOH12CR2; FAM66C; LINC00937
Chr 14	Chr14:p13	SNHG10
Chr 15	Chr15:p13	LINC00923
Chr 17	Chr17:p13.3	SNHG20; LINC00910
Chr 18	Chr18:p11.32	LINC00470; LINC00667
Chr 19	Chr19:p13.3	LINC00662
Chr 20	Chr20:p13	LINC00493; SNHG17; SNHG11
Chr 22	Chr22:p13	CECR7; DGCR5; TUG1; LINC00634

**Table 4:** Character of lncRNAs in LUAD

Chromosome	Cytoband	LncRNA
Chr 1	Chr1:p11.2	LINC00623
	Chr1:q21.2	LINC00624
	Chr1:q21.3	FALEC; ADAMTSL4-AS1; LINC00302
	Chr1:q24.2	LINC00626
	Chr1:q25.1	GAS5
	Chr1:p36.33	MIR205HG
	Chr1: q32.2	LINC00467
Chr 3	Chr3:p26.3	IGF2BP2-AS1
Chr 5	Chr5:p15.2	LINC01194
	Chr5:p13.1	LINC00603
	Chr5:p15.33	EXOC3-AS1
Chr 6	Chr6:p25.3	LINC01600
	Chr6:p22.1	HCG18
Chr 7	Chr7:p14.1	LINC00265
	Chr7:p13;	LINC00957; SNHG15
	Chr7:q21.12	TP53TG1
Chr 8	Chr8:q22.3	BAALC-AS2
	Chr8:q24.13	CASC8
	Chr8:q24.21	PVT1
	Chr8:q24.22	HPYR1
	Chr8:q13.1	SNHG6
Chr 9	Chr9:p21.3	MIR31HG; CDKN2A-AS1;
Chr12	Chr12:q14.1	LINC01465
Chr 14	Chr14:q13.2	LINC00609; PTCSC3
	Chr14:q21.1	LINC00517
Chr 17	Chr17:q25.3	SNHG20
Chr 19	Chr19:q11	LINC00662
Chr 20	Chr20:q12	SNHG17
	Chr20:q13.13	ZFAS1
	Chr20:p13	HAR1A
Chr X	ChrX:q28	LINC00894

### GO and pathway analysis of lncRNAs with alteration frequencies > 10%

**Figure 2:**
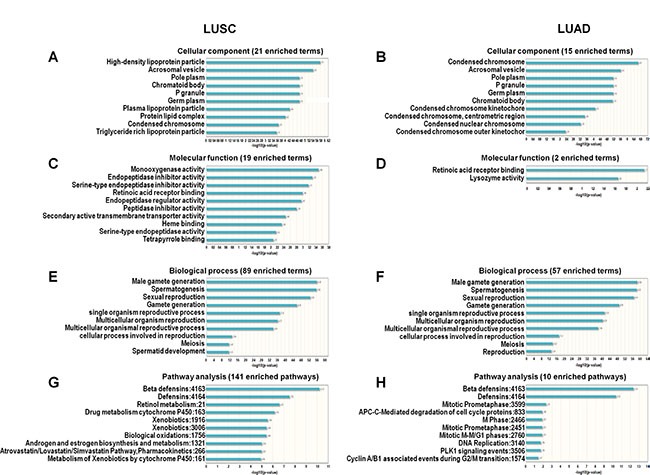
GO and pathway analysis of lncRNAs with alteration frequencies > 10% in LUSC and LUAD Cellular components enriched in LUSC (**A**) and LUAD (**B)**. Molecular functions enriched in LUSC (**C**) and LUAD (**D**). Biological processes enriched in LUSC (**E**) and LUAD (**F**) Pathways enriched in LUSC (**G**) and LUAD (**H)**.

The predicted functions of lncRNAs with alteration frequencies > 10% were explored using computational bioinformatics (*LncRNA2Function* v70 (GENCODE v15) http://mlg.hit.edu.cn/lncrna2function/index.jsp). In GO analysis, top ten cell component analysis showed five overlapped components: acrosomal vesicle, pole plasm, p granule, germ plasm, chromatoid body (Figure 2A–2B). Molecular function analysis showed only two enriched terms (retinoic acid receptor binding and lysozyme activity) were found in the LUAD lncRNA list. Retinoic acid receptor binding function was common for LUSC and LUAD (Figure 2C–2D). Even though there were cellular component and molecular function differences between LUSC and LUAD, the top ten biological processes were identical for both lung cancer types (Figure 2E–2F). Similarly, the top two pathways were the same between LUSC and LUAD: beta defensins and defensins (Figure 2G–2H).

### LncRNAs predict overall survival in LUSC and LUAD

**Figure 3:**
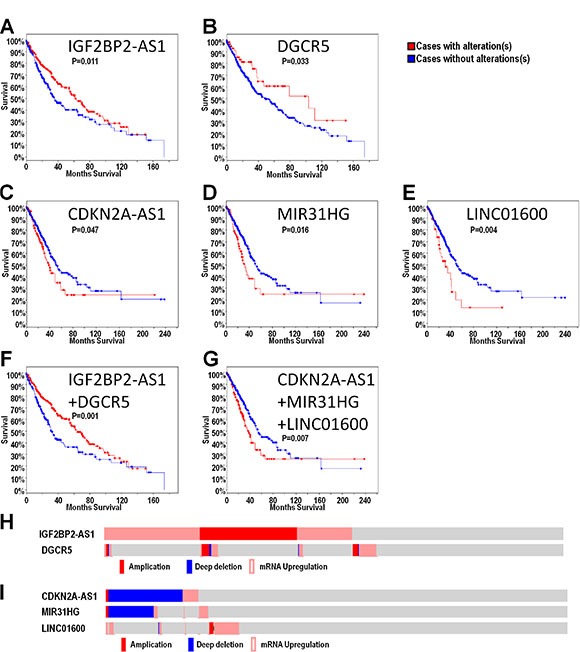
LncRNAs may predict overall survival in LUSC and LUAD Overall survival analysis was presented as a KM curve by cBioPortal. Alteration of IGF2BP2-AS1 and DGCR5 alteration may predict good prognosis in LUSC (**A**–**B**) Alteration of CDKN2A-AS1, MIR31HG and LINC01600 alterations may predict poor prognosis in LUAD (**C**–**E**) Combination of IGF2BP2-AS1 and DGCR5 alterations may predict good prognosis in LUSC (**F**). Combination of CDKN2A-AS1, MIR31HG and LINC01600 may predict poor prognosis in LUAD (**G**). Upregulation and amplification were the primary IGF2BP2-AS1 and DGCR5 alterations (**H)**. Deletion was the primary CDKN2A-AS1 and MIR31HG alteration, while LINC01600 was mainly upregulated (**I**).

cBioPortal results showed that out of all highly-altered lncRNAS, only IGF2BP2-AS1 and DGCR5 potentially predicted better overall survival in LUSC (Figure 3A–3B), and only CDKN2A-AS1, MIR31HG and LINC01600 potentially predicted poor overall survival in LUAD (Figure 3C–3E). Combinations of these lncRNAs were further analyzed. The combination of IGF2BP2-AS1 and DGCR5 in LUSC and the trio combination (CDKN2A-AS1, MIR31HG and LINC01600) in LUAD could improve patient outcome prediction capability (Figure 3F–3G). In LUSC patients, lncRNA alterations mainly included upregulation and amplification (Figure 3H). In LUAD patients, CDKN2A-AS1 and MIR31HG were mainly deleted, while LINC01600 was upregulated (Figure 3I). We also investigated the prediction value of these five lncRNAs in different genders. We found that these identified lncRNAs play their roles in different genders. Generally, for the LUSC patients, IGF2BP2-AS1 play its role in male patients. For the LUAD patients, MIR31HG and LINC01600 play their roles in female patients, while CDKN2A-AS1 play its role in male patients (Supplementary Figure S2). For the five selected lncRNAs, we also performed disease free survival analysis, we found that only IGF2BP2-AS1 could still predict better DFS in LUSC, while other lncRNAs failed to predict DFS in NSCLC (Supplementary Figure S3).

### Analysis of selected lncRNA-coexpressed genes in LUSC

**Figure 4:**
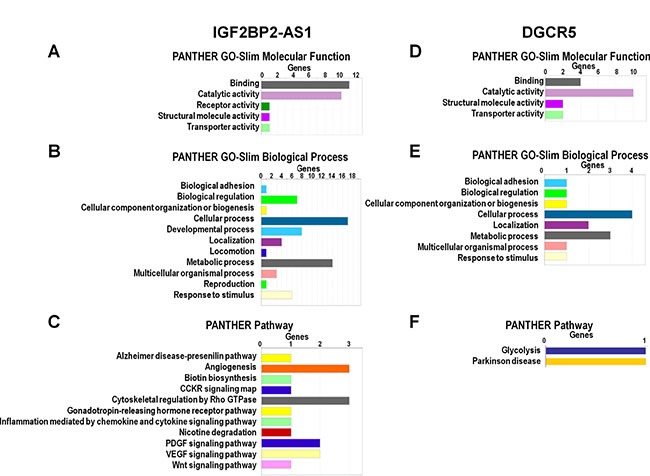
GO and pathway analysis of IGF2BP2-AS1- and DGCR5-coexpressed genes GO and pathway analysis of IGF2BP2-AS1- (**A**–**C**) and DGCR5-coexpressed (**D**–**F**) protein-coding genes in LUSC according to molecular function, biological process, and pathways based on PANTHER analysis (http://pantherdb.org/).

LncRNAs regulate genes via multiple mechanisms [14, 15]. cBioPortal analysis identified genes coexpressed with IGF2BP2-AS1, DGCR5, CDKN2A-AS1, MIR31HG and LINC01600 (Table 5). Genes coexpressed with IGF2BP2-AS1 and DGCR5 in LUSC, were analyzed in PANTHER (http://pantherdb.org/) (Figure 4). GO analysis showed that the top two enriched molecular functions for genes coexpressed with IGF2BP2-AS1 were binding and catalytic activity (Figure 4A). The most enriched biological processes were cellular process, metabolic process, developmental process, biological adhesion, and biological regulation (Figure 4B). The most enriched pathways were Alzheimer disease-presenilin pathway, angiogenesis, biotin biosynthesis, CCKR signaling map, cytoskeletal regulation by Rho GTPase, gonadotropin-releasing hormone receptor pathway, inflammation mediated by chemokine and cytokine signaling pathway, nicotine degradation, PDGF signaling pathway, VEGF signaling pathway, and Wnt signaling pathway (Figure 4B). The top two molecular functions for genes coexpressed with DGCR5 were also binding and catalytic activity (Figure 4D). The top three enriched biological processes were cellular process, metabolic process and localization (Figure 4E). The most enriched biological processes were glycolysis and Parkinson's disease (Figure 4F).

**Table 5:** Coexpression gene list of IGF2BP2-AS1, DGCR5, CDKN2A-AS1, MIR31HG, and LINC01600

LncRNA	Coexpressed gene
CDKN2A-AS1	IFNE; CDKN2A; CDKN2B; MTAP
MIR31HG	ACTN1; ANXA2; ANXA2P1; ANXA2P2; ARNTL2; BCAR3; CAMK2N1; CDC42EP2; CDCP1; CDKN2A; E2F7; EFHD2; FOSL1; FRMD6; FSCN1; GAPDH; GJB3; IFNA1; IFNA13; IFNE; LAMC2; LINC00704; MTAP; PKM; PLCD3; PLEK2; PLIN3; PML; SERPINB5; SLCO4A1; TGFBI; TMEM171; TPM4; TRPA1; VEGFC; ZYX
LINC01600	SPC25; TMPO-AS1; TIMELESS
IGF2BP2-AS1	TMEM246; CCDC136; SND1-IT1; DACT2; FAXC; C6ORF10; LOC730101; FBXL21; FBN2; ARHGEF28; KCNIP4-IT1; ETV5; IGF2BP2; SENP2; TMEM41A; CLCN2; FAM131A; YEATS2; DVL3; SOX2; TRA2B; B4GALT4; MAGEF1; TRAK1; UGT1A5; ARHGAP8; PRR5-ARHGAP8; CRYBB3; PAK7; C17ORF82; ETV4; BLMH; LOC646214; SPTLC2; SOX21; DGKA; NAB2; DTX3; GPRC5D; TSPAN18; KC6
DGCR5	SEPT5; DGCR9; RTN4R; DGCR10; EBF4; DIRAS1; ATP8B3; ENO2

### Analysis of selected lncRNA-coexpressed genes in LUAD

**Figure 5:**
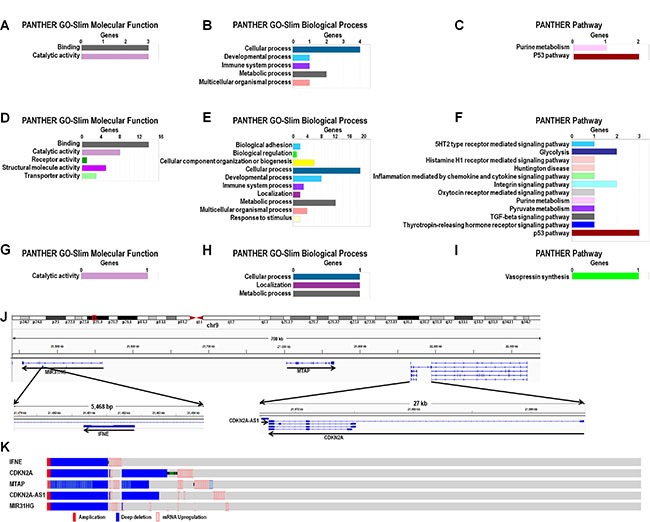
GO, pathway, and genomic analysis of CDKN2A-AS1, MIR31HG and LINC01600-coexpressed genes Enrichment analysis of GO terms and pathways for CDKN2A-AS1- (**A**–**C**) MIR31HG- (**D**–**F**)., and LINC01600-coexpressed (**G**–**I**) protein-coding genes in LUAD based on PANTHER (http://pantherdb.org/). CDKN2A-AS1, MIR31HG and MTAP genomic positions (**J**) Main IFNE, CDKN2A, MTAP, MIR31HG and CDKN2A-AS1 alterations (**K)**.

Genes coexpressed with CDKN2A-AS1, MIR31HG and LINC01600 were analyzed. The most important molecular functions for both CDKN2A-AS1- and MIR31HG-coexpressed genes were binding and catalytic activity (Figure 5A and 5D). The top two enriched biological pathways were cellular process and metabolic process (Figure 5B and 5E), and the most enriched pathway was the P53 pathway (Figure 5C and 5F). GO and pathway analysis of LINC01600-coexpressed genes showed that catalytic activity was the only enriched molecular function (Figure 5G). Cellular process, localization and metabolic process were the primary biological functions (Figure 5H), and vasopressin synthesis was the only enriched pathway (Figure 5I).

CDKN2A-AS1 and MIR31HG are not far from one another on the same chromosome (Figure 5J), and three coexpressed genes, again on the same chromosome, were common to both: IFNE, CDKN2A and MTAP. cBioPortal analysis results showed that all three coexpressed genes shared very similar alteration patterns with CDKN2A-AS1 and MIR31HG (Figure 5K). MTAP was the only gene located between CDKN2A-AS1 and MIR31HG. CDKN2A was next to, and partially overlapped with, CDKN2A-AS1, and IFNE was located in the MIR31HG intron (Figure 5K).

### Regulation of highly expressed lncRNAs

**Figure 6:**
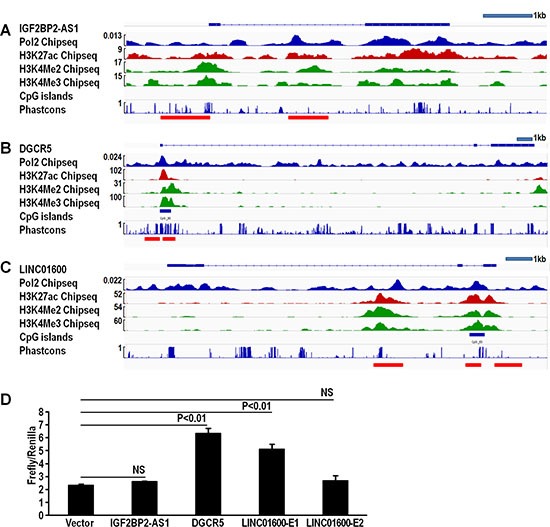
IGF2BP2-AS1, DGCR5, and LINC01600 promoter and enhancer analysis Chip-seq, CpG island, and *conservation score* analysis of IGF2BP2-AS1 promoter and enhancer; one potential enhancer was found via Chip-seq peaks (**A**). Chip-seq, CpG island, and *conservation score* analysis of DGCR5 promoter and enhancer; one potential enhancer was found via Chip-seq peaks, CpG island and high conservation score (**B**). Chip-seq, CpG island, and *conservation score* analysis of LINC01600 promoter and enhancer; two potential enhancers were found, one via Chip-seq peaks, CpG island and high conservation score (E1), and the second via Chip-seq peaks alone (E2) (**C)**. Relative luciferase activity of IGF2BP2-AS1, DGCR5, and LINC01600 promoter and enhancer (**D)**. DGCR5 promoter and enhancer, and LINC01600 promoter and enhancer 1 showed increased relative luciferase activity (triplicates, mean ± SEM). Data represented three independent experiments with similar results.

LncRNAs are regulated through regulatory regions similar to protein-coding genes, and these regulatory regions can be predicted by chip-seq analysis. IGF2BP2-AS1, DGCR5, and LINC01600 alterations were due to upregulation, so we evaluated how these lncRNAs were regulated using the ENCODE project database. There are many chip-seq datasets specific to the A549 cell line in the ENCODE database, including Pol II, H3K4Me2, H3K4Me3, and H3K27ac chip-seq data. Peaks in these data could help identify potential lncRNA promoters and enhancers [16]. CpG island hypomethylation is associated with gene overexpression [17], and conserved sequences also indicate regulatory regions [18]. We analyzed these predictors using IGV software. Promoters and potential enhancers of the three LUAD-associated lncRNAs were identified (Figure 6A–6C). Promoters were defined as 1 kb upstream of the first exon. Results identified one potential enhancer in IGF2BP-AS1 and DGCR5 (Figure 6A–6B), and two potential enhancers in LINC01600 (Figure 6C). CpG island analysis showed CpG islands in IGF2BP2-AS1 and DGCR5 promoter and enhancer regions (Figure 6A–6B). Conserved regions are more likely to be regulated and functionally important. Conservation score analysis showed that DGCR5 promoter and putative enhancer regions were highly conserved (Figure 6B). Promoter and enhancer activities were determined via luciferase reporter assay. The combination of DGCR5 promoter and putative enhancer regions exhibited increased luciferase activity. The combination of promoter and enhancer 1, but not enhancer 2 in LINC01600 drove luciferase activity. However, the combination of IGF2BP2-AS1 promoter and putative enhancer induced no luciferase activity (Figure 6D).

## DISCUSSION

LncRNAs reportedly play essential roles in lung cancer and other cancers, and may be useful biomarkers for identifying high- and low-risk patients, and predicting overall survival [19, 20]. In this study, we comprehensively analyzed highly altered lncRNAs via cBioPortal with data sourced from the TCGA database. We identified 624 lncRNAs in LUSC and 625 in LUAD with alteration frequencies > 1%, and 44 lncRNAs in LUSC and 36 in LUAD with alteration rates > 10%. A Venn diagram analysis showed that 96% of lncRNAs with alteration rates > 1%, and 20 total lncRNAs with alteration rates > 10% were shared between LUSC and LUAD. GO and pathway analyses shed light on both shared and unique lncRNA functions in LUSC and LUAD. IGF2BP2-AS1 and DGCR5 were correlated with better prognosis in LUSC, while CDKN2A-AS1, MIR31HG and LINC01600 predicted poor prognosis in LUAD. GO and pathway analyses of genes coexpressed with these lncRNAs suggested possible mechanisms by which lncRNAs could impact prognosis. IGF2BP2-AS1, DGCR5 and LINC01600 were most frequently upregulated, and we showed that regulation occurred through promoters and enhancers, similar to protein-coding genes (Supplementary Figure S1).

LncRNAs with alteration frequencies > 10% in LUSC accumulated within several cytobands. There were 22 lncRNAs in ch3: p26.3 and 10 in ch8: p23.3. Genes in these two regions might play important roles in LUSC, but not LUAD. There were few studies to investigate the relationship between cytoband and NSCLC. We found that there was a deep deletion in these cytobands. So we hypothesize that some risk factors (such as smoking) might cause the chromosome damage and thus deep deletion. This hypothesis needed further investigation. We failed to find out specific references for this finding. We found that there was a deep deletion in these cytobands. So we hypothesize that some risk factors (such as smoking) might cause the chromosome damage and thus deep deletion. These cytobands might be key regions for LUSC. There was no such accumulation observed in LUAD. While the functions of some specific lncRNAs have been investigated [21–24], the overall function of highly altered lncRNAs in NSCLC was still unclear. In this study, we used LncRNA2Function, a web-based software, to perform GO and pathway analysis [25], which revealed many similarities between LUSC and LUAD. In cell component analysis, both LUSC and LUAD shared condensed chromosome, acrosomal vesicle, pole plasm, P granule, germ plasm, and chromatoid body. The distinct LUSC cellular components were high-density lipoprotein particle, lipoprotein particle, protein lipid complex, and triglyceride rich lipoprotein particle. The cellular components unique to LUAD were condensed chromosome kinetochore, condensed chromosome, centrometric region, condensed nuclear chromosome, and condensed chromosome outer kinetochor. There were only two enriched molecular function terms (retinoic acid receptor binding and lysozyme activity) in LUAD. *Retinoic acid receptor* beta is a tumor suppressor, and a prognostic indicator in stage I NSCLC [26]. Our results showed that lncRNAs in both LUSC and LUAD shared the retinoic acid receptor binding molecular function, which could be a target of these lncRNAs. The top nine enriched biological process terms were the same between LUSC and LUAD: male gamete generation, spermatogenesis, sexual reproduction, gamete generation, single organism reproductive process, multicellular organism reproduction, multicellular organismal reproductive process, cellular process involved in reproduction, and meiosis. All these terms, except reproduction, fell under the multicellular organism reproduction term. Female and male LUSC and LUAD patients experience differential pathogeneses and prognoses, and reproduction and sexual hormones can affect lung cancer outcome. A prospective study investigated the associations between reproductive factors, hormone use, and lung cancer risk, and found that both endogenous and extraneous estrogen may drive pathogenesis [27]. Our results suggest that lncRNAs might play roles in reproduction, thus modifying NSCLC pathogenesis and prognosis. Detailed overall survival analysis by gender could help determine the roles of lncRNAs in NSCLC. In our study, we found that for the LUSC patients, IGF2BP2-AS1 play its role in male patients. For the LUAD patients, MIR31HG and LINC01600 play their roles in female patients, while CDKN2A-AS1 play its role in male patients. A bunch of lncRNAs have been shown to participate in NSCLC.

In pathway analyses, LUSC and LUAD shared the top two pathways: beta defensins and defensins. Beta defensins were detected in lung epithelial cells and epithelial cell-derived lung cancer cells. Serum beta defensin level could be a lung cancer diagnostic marker [26]. LncRNAs might influence both LUSC and LUAD through beta defensins pathway. LncRNAs could also affect LUSC or LUAD through other distinct pathways. The other eight pathways in LUSC were retinol metabolism, drug metabolism cytochrome P450, xenobiotics, biological oxidations, androgen and estrogen biosynthesis and metabolism, Atrovastatin/Lovastatin/Simvastatin pathway, pharmacokinetics, and metabolism of xenobiotics by cytochrome P450. Retinoid might be associated with LUSC, but this needs further confirmation [28]. Cytochrome P450 was associated with cancer development, and polymorphisms are lung cancer risk factors [29]. Other pathways were also related to lung cancer development [30–34]. The remaining pathways in LUAD included mitotic prometaphase, APC-C-mediated degradation of cell cycle proteins, M phase, mitotic prometaphase, mitotic M-M/G1 phases, DNA replication, PLK1 signaling events, and cyclin A/B1 associated events during G2/M transition. All of these pathways have been associated with lung cancer [35–39].

In the present study, the overall survival was analyzed with respect to the NSCLC lncRNA landscape. IGF2BP2-AS1 and DGCR5 upregulation and amplification predicted better LUSC prognosis, possibly functioning as tumor suppressors. IGF2BP2-AS1 is a lncRNA located in the *IGF2BP2* antisense strand. IGF2BP2 promotes many cancer types [40–42], and IGF2BP2-AS1 might regulate *IGF2BP* expression, thus suppressing LUSC development and progression. DGCR5 is a DiGeorge syndrome-related gene [43], and its role in cancers is currently uncertain. MIR31HG, CDKN2A-AS1 and LINC01600 predicted poor overall survival in LUAD. MIR31HG and CDKN2A-AS1 may act as tumor suppressors. MIR31HG promotes pancreatic ductal adenocarcinoma growth [44] and its downregulation was associated with bladder cancer development. In this study, we found that MIR31HG copy number deletion predicted poor prognosis in LUAD. CDKN2A-AS1 is an intronic lncRNA in the *CDKN2A* antisense strand, and is a tumor suppressor in NSCLC [45–47]. So it is very possible that CDKN2A-AS1 could regulate CDKN2A to affect lung cancer development. It seemed incompatible as for CDKN2A-AS1 and MIR31HG. We hypothesize that these two lncRNAs might help maintain the anti-cancer microenviroment or other similar mechanism. LINC01600 is an intergenic non-protein coding RNA, and very few studies have investigated its role in cancers.

LncRNAs can regulate proximal gene expression as well as that of genes farther away. We identified and investigated genes coexpressed with IGF2BP2-AS1, DGCR5, MIR31HG, CDKN2A-AS1 and LINC01600. GO-slim biological process analysis showed that both IGF2BP2-AS1 and DGCR5-coexpressed genes clustered within biological adhesion, biological regulation, cellular component organization or biogenesis, cellular process, localization, metabolic process, multicellular organismal process, and response to stimulus. IGF2BP2-AS1 and DGCR5 might play roles during LUSC development through similar biological processes. In pathway analysis, we found that IGF2BP2-AS1-coexpressed genes participated in many pathways already associated with LUSC, such as angiogenesis [48], biotin biosynthesis [49], CCKR signaling map [50], cytoskeletal regulation by Rho GTPase [51], gonadotropin-releasing hormone receptor pathway [52], inflammation mediated by chemokine and cytokine signaling [53], nicotine degradation [53], PDGF signaling [54], VEGF signaling [55], and Wnt signaling [56]. There were only two enriched pathways associated with the DGCR5-coexpressed genes: glycolysis and Parkinson's disease. Glycolysis is already associated with NSCLC development and prognosis [57, 58].

*MIR31HG* and *CDKN2A-AS1* are on the same chromosome. Three genes were located between these two lncRNA genes: *IFNE*, *MTAP*, and *CDKN2A*, all of which are associated with cancer [46, 59, 60]. MIR31HG is a lncRNA that may be a tumor suppressor in glioblastoma [61]. MIR31HG downregulation promotes cell proliferation and was correlated with poor prognosis in gastric cancer [62]. In this study, we found that *MIR31HG* deletion predicted poor prognosis in LUAD.

LncRNA regulation mechanisms are still unclear, but they may be regulated similarly to protein-coding genes [15, 63]. Previous studies showed that actively transcribed lncRNAs could be marked by RNA polymerase II (Pol II) and trimethylation of lysine 4 of histone H3 (H3K4me3) [64]. Dimethylation of histone H3 at lysine 4 (H3K4Me2) helped predict promoters and enhancers [65], and H3K27ac helped identify active enhancers [66]. CpG island and conservation analysis could also help to identify regulatory regions [17, 18]. Chip-seq data from the ENCODE project [16] were viewed in IGV software [67], integrating both CpG island data and conservation scores. In this study, we identified putative enhancers of IGF2BP2-AS1, DGCR5, and LINC01600. We confirmed that the DGCR5 and LINC01600 promoters and enhancers exhibited elevated activity in a luciferase reporter assay. This suggests that transcription factors could bind to these regulatory regions to direct their expression, thus affecting lung cancer development.

This study had several limitations. First, data were sourced from TCGA database and analyzed with cBioPortal. Even though there were more than 500 samples in both the LUAD and LUSC groups, our analyses may have missed potential biomarkers due to sample number restrictions. Similarly, we were unable to study some TCGA lncRNAs, because some were not recognized by cBioPortal. Additionally, because we chose the provisional database, database updates might affect our results and conclusions. Second, most patients in the TCGA database were white, and our study conclusions may have been different had we had access to Asian and African patient data. Third, lncRNA GO and pathway analyses were based on LncRNA2Function. LncRNA2Function is based on expression correlations between lncRNAs and protein-coding genes across 19 human normal tissues [25], and there might have been some false positive and negative function predictions. Fourth, lncRNA mechanisms of action in lung cancer development should be confirmed by knockout in lung cancer cells. Transgenic mice lacking these specific lncRNAs could also provide further functional information. Fifth, the transcription factors that regulate lncRNAs must still be identified.

In conclusion, this study presented a lncRNA landscape in NSCLC, and identified differentially expressed, highly altered lncRNAs in LUSC and LUAD. Two lncRNAs (IGF2BP2-AS1 and DGCR5) were correlated with better survival in LUSC and three (CDKN2A-AS1, MIR31HG, and LINC01600) could predict poor prognosis in LUAD. GO and pathway analysis of highly altered lncRNAs and their coexpressed genes helped identify potential mechanisms of action in lung cancer pathogenesis. Chip-seq and luciferase reporter analysis of the selected lncRNAs showed that they could be regulated through promoters and enhancers, similar to protein-coding genes. LncRNAs that act as oncogenes in NSCLC, and transcription factors that regulate cancer-related lncRNAs provide potential novel targets for anti-cancer therapeutics.

## MATERIALS AND METHODS

### Cell culture

The lung cancer cell line, A549, was cultured in the Iscove's Modified Dulbecco's Medium (Corning, Manassas, VA) with 10% fetal bovine serum (FBS) (Fisher, Grand Island, NY), 100 units/mL penicillin and 100 μg/mL streptomycin. Cells were subcultured every two to three days. Confluence was maintained between 20% and 70%.

### LncRNA analysis

The lncRNA list was downloaded from the HUGO gene nomenclature committee (HGNC) (http://www.genenames.org/). 2745/2772 lncRNAs from the HUGO database were recognized by cBioPortal. There were 504 LUSC (“Lung Squamous Cell Carcinoma (TCGA, Provisional) 504 samples” dataset) and 522 LUAD (“Lung Adenocarcinoma (TCGA, Provisional) 522 samples” dataset) samples in the TCGA database (TCGA, Provisional). LncRNA somatic mutations, DNA copy number alterations (CNAs), and expression values were integrated into cBioPortal. LncRNA alteration frequency was defined as the number of cases with lncRNA alterations divided by the total number of cases. The primary goal was to identify the lncRNA alternation rate in these samples. LncRNAs with alternation rates > 10% were further analyzed to identify correlations with overall survival. Genes coexpressed with the selected lncRNAs were also identified by cBioPortal.

### Gene ontology (GO) and pathway analysis

LncRNA2Function is an ontology-driven tool for exploring potential lncRNA functions to guide experimental and clinical investigations [25]. It is based on expression correlations between lncRNAs and protein-coding genes across 19 human normal tissues. LncRNAs with alteration frequencies > 10% were analyzed with this tool for GO and human biological pathways. The PANTHER (Protein ANalysis THrough Evolutionary Relationships) classification system was used to identify enriched GO terms and biological pathways for coexpressed genes.

### IGV-facilitated chromatin immunoprecipitation followed by sequencing (chip-seq), and LncRNA CpG island and conservation score analysis

The Encyclopedia of DNA Elements (ENCODE) has constructed a comprehensive list of functional human genome elements, including those that act at the RNA level, and regulatory elements that control gene transcription (https://www.encodeproject.org/) [16]. We choose the GSE33213 series for the Pol II chip-seq data and the GSE29611 series for the H3K4me2, H3K4me3 and H3K27ac chip-seq data in the A549 cell line. The Integrative Genomics Viewer (IGV) is a high performance visualization tool that has integrated the ENCODE database and human assemblies, such as CpG islands and various conservation tracks [67]. For the chip-seq analysis, we loaded the ENCODE data into the IGV software. For the CpG island analysis, we chose “load from server” in the file option, then “annotations-sequence and regulation-CpG islands.” For the conservation score analysis, we chose “load from server” in the file option, then “annotations-comparative genomics-Phastcons (vertebrate 46 way).”

### Vector construction and transfection, and luciferase reporter assay

LncRNA IGF2BP2-AS1, DGCR5, and LINC01600 promoters and enhancers were cloned into the PGL3 vector. With specific primers (Supplementary Table S5) 20 μg DNA from A549 cells was used as a PCR template. For IGF2BP2-AS1 promoter and enhancer construction, we first PCR-amplified the promoter region, then digested it with NheI and XhoI for 1 h at 37°C. Digested DNA fragments were inserted into the similarly-digested PGL3 vector. Then we PCR-amplified the enhancer region, and digested it with KpnI and MluI. Digested DNA fragments were cloned into the PGL3 vector containing the IGF2BP2-AS1 promoter. The DGCR5 promoter and enhancer were cloned into the PGL3 vector via a similar procedure using BglII and HindIII for promoter construction, and MluI and XhoI enhancer construction. NheI and XhoI were used for LINC01600 promoter construction, and KpnI and MluI for enhancer construction. Plasmids were purified with *Endo-Free Plasmid* Maxi *Kits (Omega Bio-tek*). For transient transfection, 5×10^5^ A549 cells/well were subcultured in 6-well plates for 18 h. Medium containing penicillin/streptomycin in wells was replaced before transfection. Two μg PGL3-target firefly plasmid and 0.2 μg PRL-SV40 renilla plasmid were added to 200 μl cell culture medium (without FBS and penicillin/streptomycin). 6.6 μg *polyethylenimine* (PEI) was added into the solution and mixed gently by pipette for 10 s. The DNA-PEI-medium mixture was incubated at room temperature for 20 min, then added to wells. The 6-well plate was swirled gently for 10 s. Six h later, the solution was removed from wells and fresh medium was added. Twenty-four h after transfection, luminescence was measured using the Dual-Luciferase^®^ Reporter (DLR™) Assay System (Promega) following the manufacture's protocol.

### Statistical analysis

All valid lncRNAs identified by cBioPortal were sorted by alteration frequency. LncRNAs with alternation frequency > 10% were selected for further analysis. To determine lncRNA alternation frequency differences between LUSC and LUAD, *venn* diagram analysis was performed (http://bioinformatics.psb.ugent.be/webtools/Venn/). Kaplan-Meier analysis was performed with cBioPortal to determine overall survival. Student's *t*-test was used to determine statistical differences between two groups. *P* < 0.05 was considered statistically significant.

## SUPPLEMENTARY MATERIALS FIGURES AND TABLES










