# Supplementary material for: LncRNAs are altered in lung squamous cell carcinoma and lung adenocarcinoma
Source: Oncotarget. 2016 Nov 26;8(15):24275–91. doi: 10.18632/oncotarget.13651 (PMC5421846; doi:10.18632/oncotarget.13651)
Supplement: Supplementary file 1 [file oncotarget-08-24275-s001.pdf]

LncRNAs are altered in lung squamous cell carcinoma and lung adenocarcinoma

Supplementary Materials

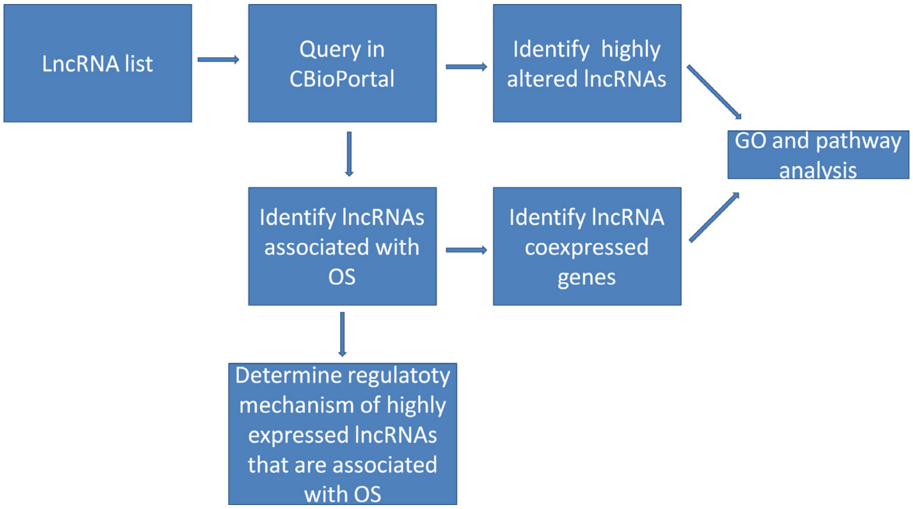

Supplementary Figure S1: Flowchart indicating how lncRNAs were analyzed in this paper. The lncRNA list was downloaded from HUGO, and was queried in cBioPortal software. Highly altered lncRNAs and lncRNAs associated with overall survival (OS) wereidentified. Coexpressed gene lists were generated through cBioPortal. GO and pathway analysis were performed for highly altered lncRNAs and coexpressed genes. For lncRNAs associated with OS, potential promoters and enhancers were predicted, and their activities analyzed.

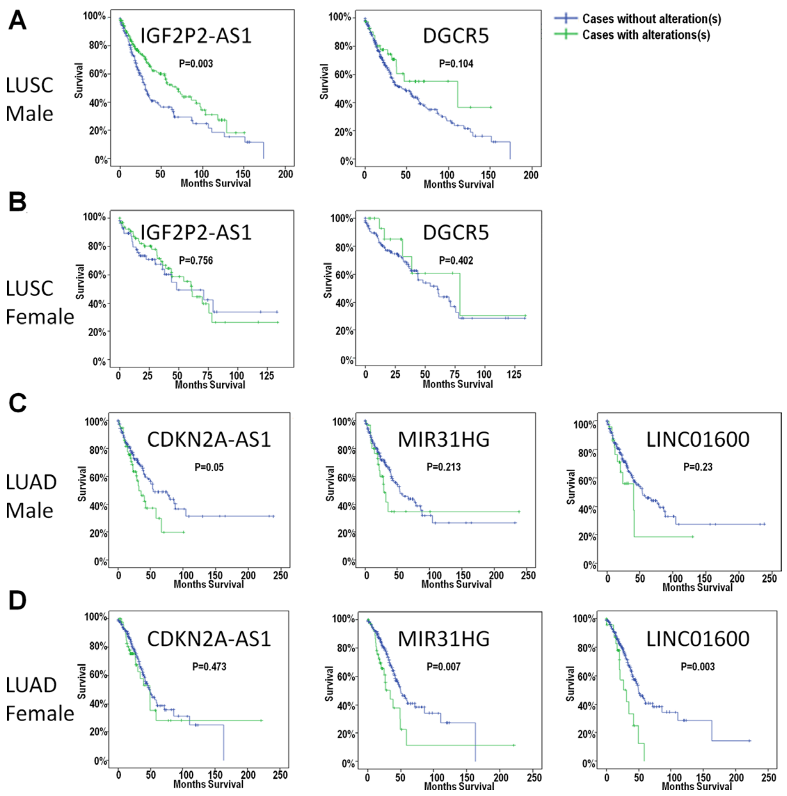

Supplementary Figure S2: Overall survival analysis of the five selected lncRNAs grouped by gender. For the five selected lncRNAs, the overall survival data and clinical information were downloaded from cBioPortal website. Overall survival of these lncRNAs were analyzed between different genders. (**A**) Overall survival analysis of IGF2BP2-AS1 and DGCR5 in LUSC in male patients; (**B**) Overall survival analysis of IGF2BP2-AS1 and DGCR5 in LUSC in female patients; (**C**) Overall survival analysis of CDKN2A-AS1, MIR31HG and LINC01600 in LUAD in male patients; (**D**) Overall survival analysis of CDKN2A-AS1, MIR31HG and LINC01600 in LUAD in female patients;

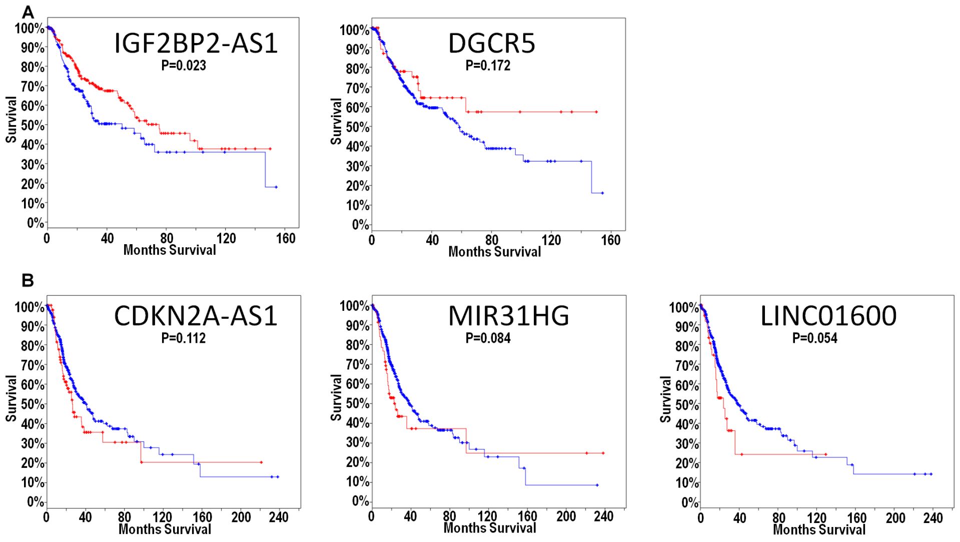

Supplementary Figure S3: Disease free analysis of the five selected lncRNAs. (**A**) Disease free analysis of IGF2BP2-AS1 and DGCR5 in LUSC; (**B**) Disease free analysis of CDKN2A-AS1, MIR31HG and LINC01600 in LUAD.

Supplementary Table S1: LncRNA downloaded from HUGO. See Supplementary_Table_S1

Supplementary Table S2: LncRNAs valid in cBioPortal. See Supplementary_Table_S2

Supplementary Table S3: LncRNAs with alteration frequency higher than 1% in LUSC. See Supplementary_Table_S3

Supplementary Table S4: LncRNAs with alteration frequency higher than 1% in LUAD. See Supplementary_Table_S4

Supplementary Table S5: Primer sequences for plasmid construction

Primer	Sequence
IGF2BP2AS1Promoter-F	AAATGCTAGCGAAATGTAAAATTTGCAATAATC
IGF2BP2AS1Promoter-R	AATACTCGAGGTTATGGGCCAGAGTTCTGT
IGF2BP2AS1Enhancer-F	AAATGGTACCGTTAGATACCAGAAAGACAGCAT
IGF2BP2AS1Enhancer-R	AATAACGCGTGAACCTGGAAGGTGGAGG
DGCR5Promoter-F	AATAAGATCTTCTGCTTCATATCAGTGGACCT
DGCR5Promoter-R	AAATAAGCTTCCCAGGTCCACTGATATGAA
DGCR5Enhancer-F	AATAACGCGTGGTGAGTGTCCACGCGGC
DGCR5Enhancer-R	AAATCTCGAGGTGCAGCCTGCGACACCA
LINC016000Promoter-F	GGCGGCTAGCGTACACGTTTTCATATTTAATTA
LINC016000Promoter-R	AATACTCGAGTGCAGCGCGGCGTGGGCC
LINC01600Enhancer1-F	AAATGGTACCCGTGTCTTTCTTATCAAGCC
LINC01600Enhancer1-R	AATAACGCGTAAGCTCTGTTATTATTATCTTTTG
LINC01600Enhancer2-F	AAATGGTACCATGACCTGTTTTTCCTGACTC
LINC01600Enhancer2-R	AATAACGCGTTTCTCTTTCCCTACTCCCC
