# Supplementary material for: LncRNAs are altered in lung squamous cell carcinoma and lung adenocarcinoma
Source: Oncotarget. 2016 Nov 26;8(15):24275–91. doi: 10.18632/oncotarget.13651 (PMC5421846; doi:10.18632/oncotarget.13651)
Supplement: Supplementary file 2 [file oncotarget-08-24275-s002.docx]

Table 1 LncRNAs with alteration frequency higher than 10% in LUSC

| **LncRNA** | **Alteration** | **Alteration reason** | **Cytoband** | **Position** |
| --- | --- | --- | --- | --- |
| IGF2BP2-AS1 | 0.54 | Amp; high exp | p26.3 | Ch3:185,712,528-185,729,787 |
| LINC00888 | 0.46 | Amp | p26.3 | Ch3:183,447,608-183,456,013 |
| LINC00501 | 0.44 | Amp | p26.3 | Ch3:177,294,442-177,323,418 |
| LINC00578 | 0.44 | Amp | p26.3 | Ch3:177,441,921-177,752,305 |
| LINC00887 | 0.40 | Amp; high exp | p26.3 | Ch3:194,296,465-194,312,803 |
| LINC00884 | 0.38 | Amp | p26.3 | Ch3:194,487,454-194,518,279 |
| LINC00969 | 0.38 | Amp | p26.3 | Ch3:195,658,062-195,739,964 |
| LINC00885 | 0.38 | Amp | p26.3 | Ch3:196,142,636-196,160,890 |
| LINC01192 | 0.35 | Amp | p26.3 | Ch3:163,127,923-163,361,563 |
| EXOC3-AS1 | 0.33 | Amp; high exp | p15.33 | Ch5:441,498-443,160 |
| LINC00880 | 0.29 | Amp | p26.3 | Ch3:157,081,667-157,123,004 |
| LINC00881 | 0.29 | Amp | p26.3 | Ch3:157,089,881-157,101,135 |
| CDKN2A-AS1 | 0.29 | Del | p24.3 | Ch9:21,966,929-21,967,751 |
| LINC00886 | 0.28 | Amp | p26.3 | Ch3:156,747,346-156,817,062 |
| PVT1 | 0.23 | High exp; amp | p23.3 | Ch8:127,794,533-128,101,253 |
| BPESC1 | 0.2 | Amp; high exp | p26.3 | Ch3:139,104,185-139,125,171 |
| DUBR | 0.18 | Amp; high exp | p26.3 | Ch3:107,240,692-107,326,964 |
| LINC00635 | 0.18 | Amp; high exp | p26.3 | Ch3:107,840,228-107,882,000 |
| LINC01194 | 0.18 | Amp | p15.33 | Ch5:12,574,857-12,804,363 |
| MIR31HG | 0.17 | Del; high exp | p24.3 | Ch9:21,455,642-21,559,669 |
| TUG1 | 0.16 | Amp; del | p13 | Ch22:30,970,677-30,979,395 |
| LINC01565 | 0.16 | Amp; high exp | p26.3 | Ch3:128,572,000-128,576,086 |
| CASC8 | 0.16 | Amp; high exp | p23.3 | Ch8:127,289,817-127,482,139 |
| LINC00603 | 0.15 | Amp | p15.33 | Ch5:40,052,291-40,053,324 |
| TUSC7 | 0.14 | Amp; high exp | p26.3 | Ch3:116,709,235-116,723,581 |
| LINC00964 | 0.14 | High exp; amp | p23.3 | Ch8:124,848,737-124,954,328 |
| LINC00623 | 0.13 | High exp; amp | p36.33 | Ch1:120,913,275-121,009,291 |
| SNHG20 | 0.13 | Amp; high exp | p13.3 | Ch17:77,086,716-77,094,990 |
| SNHG11 | 0.13 | High exp | p13 | Ch20:38,446,578-38,450,921 |
| LINC00882 | 0.13 | Amp; high exp | p26.3 | Ch3:106,836,811-107,240,641 |
| LINC00879 | 0.13 | Amp | p26.3 | Ch3:94,938,172-95,152,509 |
| HPYR1 | 0.13 | Amp; high exp | p23.3 | Ch8:132,560,498-132,561,479 |
| LINC00626 | 0.12 | High exp; amp | p36.33 | Ch1:168,786,939-168,792,886 |
| MIR205HG | 0.12 | High exp; amp | p36.33 | Ch1:209,428,820-209,432,838 |
| FAM66C | 0.12 | High exp; amp | p13.33 | Ch12:8,180,209-8,216,151 |
| DGCR5 | 0.12 | High exp; amp | p13 | Ch22:18,970,514-19,031,242 |
| LINC00488 | 0.12 | Amp; high exp | p26.3 | Ch3:109,178,165-109,185,257 |
| FAM167A-AS1 | 0.12 | Del; high exp | p23.3 | Ch8:11,368,402-11,438,658 |
| FAM95B1 | 0.12 | high exp; amp | p24.3 | Ch9:40,321,299-40,329,221 |
| GAS5 | 0.11 | high exp | p36.33 | Ch1:173,863,900-173,868,882 |
| LINC00937 | 0.11 | High exp; amp | p13.33 | Ch12:8,295,986-8,396,803 |
| SNHG10 | 0.11 | High exp; | p13 | Ch14:95,532,297-95,534,872 |
| LINC00662 | 0.11 | High exp; amp | p13.3 | Ch19:27,684,580-27,793,940 |
| SNHG17 | 0.11 | High exp; | p13 | Ch20:38,420,588-38,435,353 |
| CECR7 | 0.11 | High exp; | p13 | Ch22:17,036,570-17,060,825 |
| SNHG15 | 0.11 | High exp; | p22.3 | Ch7:44,983,023-44,986,961 |
| FAM74A3 | 0.11 | High exp; | p24.3 | Ch9:66,976,520-66,976,991 |
| LOH12CR2 | 0.1 | High exp; amp | p13.33 | Ch12:12,355,406-12,357,067 |
| LINC00923 | 0.1 | High exp; amp | p13 | Ch15:97,572,185-97,874,550 |
| LINC00470 | 0.1 | High exp; amp | p11.32 | Ch18:1,254,383-1,408,344 |
| LINC00667 | 0.1 | High exp; | p11.32 | Ch18:5,237,826-5,246,508 |
| LINC00493 | 0.1 | High exp; | p13 | Ch20:18,567,347-18,569,563 |
| LINC00634 | 0.1 | Amp | p13 | Ch22:41,952,165-41,958,933 |
| LINC00636 | 0.1 | Amp | p26.3 | Ch3:107,883,248-107,928,907 |
| LINC00901 | 0.1 | Amp | p26.3 | Ch3:116,921,431-116,932,238 |
| DANCR | 0.1 | High exp; amp | p16.3 | Ch4:52,712,404-52,720,351 |
| HCG18 | 0.1 | High | p25.3 | Ch6:30,287,397-30,327,150 |
| BAALC-AS2 | 0.1 | High amp | p23.3 | Ch8:103,132,963-103,141,475 |
| CCAT1 | 0.1 | Amp | p23.3 | Ch8:127,207,866-127,219,088 |
| LINC00051 | 0.1 | Amp; high exp | p23.3 | Ch8:142,198,356-142,209,003 |
| SNHG6 | 0.1 | High amp | p23.3 | Ch8:66,921,684-66,926,398 |
| FAM66E | 0.1 | Del; high exp | p23.3 | Ch8:7,955,014-8,008,755 |
| LINC00032 | 0.1 | Del; amp; high exp | p24.3 | Ch9:27,245,684-27,282,793 |
| LINC00910 | 0.1 | High | p13.3 | h17:43,369,845-43,389,199 |
