# Supplementary material for: LncRNAs are altered in lung squamous cell carcinoma and lung adenocarcinoma
Source: Oncotarget. 2016 Nov 26;8(15):24275–91. doi: 10.18632/oncotarget.13651 (PMC5421846; doi:10.18632/oncotarget.13651)
Supplement: Supplementary file 3 [file oncotarget-08-24275-s003.docx]

Table 2 LncRNAs with alteration frequency higher than 10% in LUAD

| **LncRNA** | **Alteration** | **Alteration reason** | **Cytoband** | **Position** |
| --- | --- | --- | --- | --- |
| EXOC3-AS1 | 0.29 | Amp; high exp | p15.33 | Chr5:441498-443160 |
| PVT1 | 0.25 | Amp; high exp | q24.21 | Chr8:127794533-128101253 |
| LINC00623 | 0.21 | Amp; high exp | p11.2 | Chr1:120912238-121009291 |
| CDKN2A-AS1 | 0.2 | Del | p21.3 | chr9:21966929-21967754 |
| LINC00467 | 0.18 | Amp; high exp | q32.2 | Chr1:211382755-211435333 |
| HCG18 | 0.15 | High exp | p22.1 | Chr6:30287397-30327156 |
| FALEC | 0.14 | Amp | q21.3 | Chr1:150515757-150518032 |
| LINC01194 | 0.14 | Amp | p15.2 | Chr5:12574857-12805183 |
| MIR31HG | 0.14 | Del | p21.3 | Chr9:21454268-21559698 |
| LINC00624 | 0.13 | Amp | q21.2 | Chr1:147258885-147517875 |
| ADAMTSL4-AS1 | 0.13 | Amp | q21.3 | Chr1:150560202-150574552 |
| LINC00302 | 0.13 | Amp | q21.3 | Chr1:152655429-152656805 |
| LINC00609 | 0.13 | Amp | q13.2 | Chr14:36070427-36165288 |
| PTCSC3 | 0.13 | Amp | q13.2 | Chr14:36135710-36176651 |
| LINC00662 | 0.13 | High exp | q11 | Chr19:27684580-27793940 |
| LINC00957 | 0.13 | Amp; high exp | p13 | Chr7:44039049-44044296 |
| LINC00626 | 0.12 | Amp; high exp | q24.2 | Chr1:168786939-168792886 |
| GAS5 | 0.12 | Amp; high exp | q25.1 | Chr1:173863900-173868882 |
| SNHG15 | 0.12 | Amp; high exp | p13 | Chr7:44983023-44986961 |
| BAALC-AS2 | 0.12 | Amp; high exp | q22.3 | Chr8:103132963-103141475 |
| LINC00517 | 0.11 | Amp | q21.1 | Chr14:37896060-37902372 |
| SNHG20 | 0.11 | High exp | q25.3 | Chr17:77086716-77094990 |
| HAR1A | 0.11 | Amp; high exp | p13 | Chr20:63102205-63104386 |
| LINC00603 | 0.11 | Amp | p13.1 | Chr5:40052291-40053324 |
| LINC00265 | 0.11 | Amp; high exp | p14.1 | Chr7:39733632-39793092 |
| CASC8 | 0.11 | Amp | q24.13 | Chr8:127289817-127482139 |
| HPYR1 | 0.11 | Amp; high exp | q24.22 | Chr8:132560498-132561479 |
| LINC01465 | 0.1 | High exp | q14.1 | Chr12:62601751-62603690 |
| SNHG17 | 0.1 | High exp | q12 | Chr20:38420588-38435353 |
| ZFAS1 | 0.1 | High exp | q13.13 | Chr20:49278178-49295738 |
| TP53TG1 | 0.1 | High exp | q21.12 | Chr7:87325225-87345515 |
| SNHG6 | 0.1 | High exp | q13.1 | Chr8:66921684-66926398 |
| LINC00894 | 0.1 | Amp; high exp | q28 | ChrX:149938628-150224580 |
| IGF2BP2-AS1 | 0.1 | High exp | p26.3 | Chr3:185712528-185729787 |
| LINC01600 | 0.1 | High exp | p25.3 | Chr6:2,621,913-2634603 |
| MIR205HG | 0.1 | Amp; high exp | p36.33 | Chr1:209428820-209432838 |
