# Supplementary material for: LncRNAs are altered in lung squamous cell carcinoma and lung adenocarcinoma
Source: Oncotarget. 2016 Nov 26;8(15):24275–91. doi: 10.18632/oncotarget.13651 (PMC5421846; doi:10.18632/oncotarget.13651)
Supplement: Supplementary file 4 [file oncotarget-08-24275-s004.docx]

Table 3 Character of lncRNAs in LUSC

| **Chromosome** | **Cytoband** | **LncRNA** |
| --- | --- | --- |
| Chr 1 | Chr1: p36.33 | LINC00623; LINC00626; GAS5; MIR205HG |
| Chr 3 | Chr3: p26.3 | LINC00882; DUBR; LINC00635; LINC00636; LINC00488; TUSC7; LINC00901; LINC01565; BPESC1; LINC00886; LINC00880; LINC00881; LINC01192; LINC00501; LINC00578; LINC00888; IGF2BP2-AS1; LINC00887; LINC00884; LINC00969; LINC00885; LINC00879 |
| Chr 4 | Chr4: p16.3 | DANCR |
| Chr 5 | Chr5: p15.33 | LINC01194; LINC00603; EXOC3-AS1 |
| Chr 6 | Chr6: p25.3 | HCG18 |
| Chr 7 | Chr7: p22.3 | SNHG15 |
| Chr 8 | Chr8: p23.3 | BAALC-AS2; FAM167A-AS1; LINC00964; CCAT1; CASC8; PVT1; HPYR1; LINC00051; SNHG6; FAM66E |
| Chr 9 | Chr9: p24.3 | MIR31HG; CDKN2A-AS1; LINC00032; FAM95B1; FAM74A3 |
| Chr 12 | Chr12: p13.33 | LOH12CR2; FAM66C; LINC00937 |
| Chr 14 | Chr14: p13 | SNHG10 |
| Chr 15 | Chr15: p13 | LINC00923 |
| Chr 17 | Chr17: p13.3 | SNHG20; LINC00910 |
| Chr 18 | Chr18: p11.32 | LINC00470; LINC00667 |
| Chr 19 | Chr19: p13.3 | LINC00662 |
| Chr 20 | Chr20: p13 | LINC00493; SNHG17; SNHG11 |
| Chr 22 | Chr22: p13 | CECR7; DGCR5; TUG1; LINC00634 |
