# Supplementary material for: LncRNAs are altered in lung squamous cell carcinoma and lung adenocarcinoma
Source: Oncotarget. 2016 Nov 26;8(15):24275–91. doi: 10.18632/oncotarget.13651 (PMC5421846; doi:10.18632/oncotarget.13651)
Supplement: Supplementary file 5 [file oncotarget-08-24275-s005.docx]

Table 4 Character of lncRNAs in LUAD

| **Chromosome** | **Cytoband** | **LncRNA** |
| --- | --- | --- |
| Chr 1 | Chr1: p11.2 | LINC00623 |
|  | Chr1: q21.2 | LINC00624 |
|  | Chr1: q21.3 | FALEC; ADAMTSL4-AS1; LINC00302 |
|  | Chr1: q24.2 | LINC00626 |
|  | Chr1: q25.1 | GAS5 |
|  | Chr1: p36.33 | MIR205HG |
|  | Chr1: q32.2 | LINC00467 |
| Chr 3 | Chr3: p26.3 | IGF2BP2-AS1 |
| Chr 5 | Chr5: p15.2 | LINC01194 |
|  | Chr5: p13.1 | LINC00603 |
|  | Chr5: p15.33 | EXOC3-AS1 |
| Chr 6 | Chr6: p25.3 | LINC01600 |
|  | Chr6: p22.1 | HCG18 |
| Chr 7 | Chr7: p14.1 | LINC00265 |
|  | Chr7: p13; | LINC00957; SNHG15 |
|  | Chr7: q21.12 | TP53TG1 |
| Chr 8 | Chr8: q22.3 | BAALC-AS2 |
|  | Chr8: q24.13 | CASC8 |
|  | Chr8: q24.21 | PVT1 |
|  | Chr8: q24.22 | HPYR1 |
|  | Chr8: q13.1 | SNHG6 |
| Chr 9 | Chr9: p21.3 | MIR31HG; CDKN2A-AS1; |
| Chr12 | Chr12: q14.1 | LINC01465 |
| Chr 14 | Chr14: q13.2 | LINC00609; PTCSC3 |
|  | Chr14: q21.1 | LINC00517 |
| Chr 17 | Chr17: q25.3 | SNHG20 |
| Chr 19 | Chr19: q11 | LINC00662 |
| Chr 20 | Chr20: q12 | SNHG17 |
|  | Chr20: q13.13 | ZFAS1 |
|  | Chr20: p13 | HAR1A |
| Chr X | ChrX: q28 | LINC00894 |
